# Substrate Temperature Dependent Surface Morphology and Photoluminescence of Germanium Quantum Dots Grown by Radio Frequency Magnetron Sputtering

**DOI:** 10.3390/ijms131012880

**Published:** 2012-10-09

**Authors:** Alireza Samavati, Zulkafli Othaman, Sib Krishna Ghoshal, Mohammad Reza Dousti, Mohammed Rafiq Abdul Kadir

**Affiliations:** 1 Ibn Sina Institute for Fundamental Science Studies, Universiti Teknologi Malaysia, Skudai, Johor 81100, Malaysia; E-Mail: alireza.samavati@yahoo.com; 2 Advanced Optical Material Research Group, Department of Physics, Faculty of Science, Universiti Teknologi Malaysia, Skudai, Johor 81100, Malaysia; E-Mails: krishnasib@gmail.com (S.K.G.); mrdphysics@gmail.com (M.R.D.); 3 Medical Implant Technology Group, Faculty of Bioscience and Medical Engineering, Universiti Teknologi Malaysia, Skudai, Johor 81310, Malaysia; E-Mail: rafiq@biomedical.utm.my

**Keywords:** Ge quantum dots, visible photoluminescence, RMS roughness

## Abstract

The visible luminescence from Ge nanoparticles and nanocrystallites has generated interest due to the feasibility of tuning band gap by controlling the sizes. Germanium (Ge) quantum dots (QDs) with average diameter ~16 to 8 nm are synthesized by radio frequency magnetron sputtering under different growth conditions. These QDs with narrow size distribution and high density, characterized using atomic force microscopy (AFM) and field emission scanning electron microscopy (FESEM) are obtained under the optimal growth conditions of 400 °C substrate temperature, 100 W radio frequency powers and 10 Sccm Argon flow. The possibility of surface passivation and configuration of these dots are confirmed by elemental energy dispersive X-ray (EDX) analysis. The room temperature strong visible photoluminescence (PL) from such QDs suggests their potential application in optoelectronics. The sample grown at 400 °C in particular, shows three PL peaks at around ~2.95 eV, 3.34 eV and 4.36 eV attributed to the interaction between Ge, GeO*_x_* manifesting the possibility of the formation of core-shell structures. A red shift of ~0.11 eV in the PL peak is observed with decreasing substrate temperature. We assert that our easy and economic method is suitable for the large-scale production of Ge QDs useful in optoelectronic devices.

## 1. Introduction

The Ge\Si(100) system among the self-assembled semiconductors nanostructure has generated intense interest since it is the simplest semiconductor hetero-epitaxial system that opened new possibilities for optoelectronic and microelectronic applications. The small electron and hole effective masses of bulk germanium (Ge) leads to a significantly larger excitonic Bohr radius (~24.3 nm) [1] implying strong quantum confinement effects in Ge nanocrystals (NCs). An enhanced quantum confinement leads to a decrease in indirect band gap transitions and relaxes the selection rules for direct band gap transitions. Thus, Ge NCs are expected to exhibit visible photoluminescence with the high quantum efficiency. The brilliant, tunable fluorescence emission of Ge quantum dots has encouraged their use as nanophotonics applications.

In recent years, extensive efforts have been made to fabricate Ge NCs using various chemical and physical methods [2–5]. Alkyl-surface functionalized Ge NCs via metal hydride reduction of nonpolar solutions of CTAB and GeI_4_ at room temperature has been produced by Veinot *et al.* [6]. Chiu *et al.* synthesized Ge nanoparticles using the sodium naphthalide reduction of GeCl_4_ under varying reaction conditions [5]. Simonsen *et al.* produced Ge NCs on Si (001) substrate using electron beam evaporation in which a Gaussian distribution of sizes from 2 to 68 nm was achieved, and the full width at half maximum (FWHM) showed increment with increasing island size [7]. Ge QDs having sizes 8 to 20 nm were prepared by Fahim *et al.* using electron beam evaporation technique, in which the root mean square (RMS) roughness is shown to be highly sensitive to the annealing temperature. Furthermore, the thermal annealing was found to strongly influence the structural, optical (~0.25 eV shift of the band gap energy) and electrical properties of Ge QDs [8].

Mestanza *et al.* measured the room temperature PL spectra of Ge nanocrystalline samples on SiO_2_ matrix by ion implantation technique and observed a broad blue violet band at around 3.2 eV (400 nm) originates from germanium-oxygen-deficient-centers. The occurrence of a weak peak around 4 eV is also reported [9]. The temperature dependent PL peak at 2.05 eV was observed by Sun *et al.* [10]. A PL red shift from 1.18 to 1.05 eV was found as a function of increasing nanoparticle size from 1.6 to 9.1 nm in the experiment of Riabinina *et al.* [11]. Three prominent PL peaks at 2.59 nm, 2.76 nm and 3.12 nm for Ge nanoparticles synthesized by the inert gas condensation (IGC) method were illustrated by Oku *et al*. The PL peaks are related to luminescence that originates from the Ge/GeO*_x_* interface and quantum size effect of Ge clusters. They indicated that the formation of the core-shell structure of Ge and Si with oxide layers is the reason for the blue shift of band gap energy [12].

In a recent communication, we have reported the details of preparation and characterization of Ge nanoislands having sizes ~50 nm to ~100 nm [13]. The island size and RMS roughness are found to increase and the number density is decreased on increasing the annealing temperature. The knowledge of the size and shape distribution is a prerequisite in the understanding of the evolution of islands during growth and predicting the optical properties and surface morphology of such islands. In this paper, we present the size distribution and surface evolution pattern of Ge nanoislands on Si (100) recorded using atomic force microscopy (AFM) and field emission scanning electron microscopy (FESEM). The details of the growth behavior and optical properties are investigated by PL spectroscopy with varying substrate temperature.

## 2. Results and Discussion

### 2.1. FESEM Results

**Figure 1:**
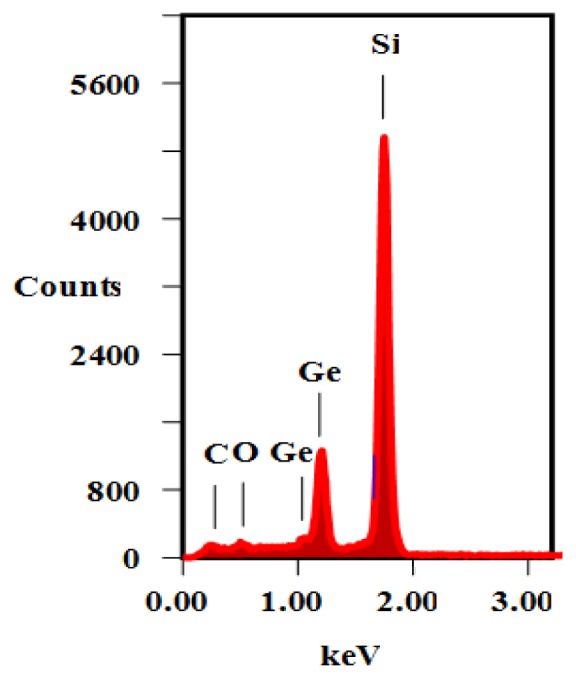
Energy dispersive X-ray (EDX) spectra of sample D.

**Figure 2:**
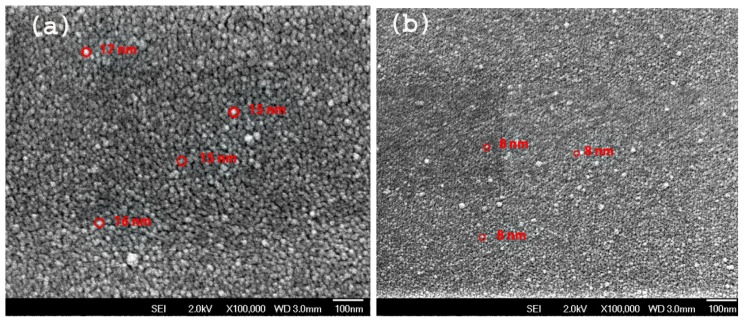
Field emission scanning electron microscopy (FESEM) image of sample A (**a**) and D (**b**).

**Figure 3:**
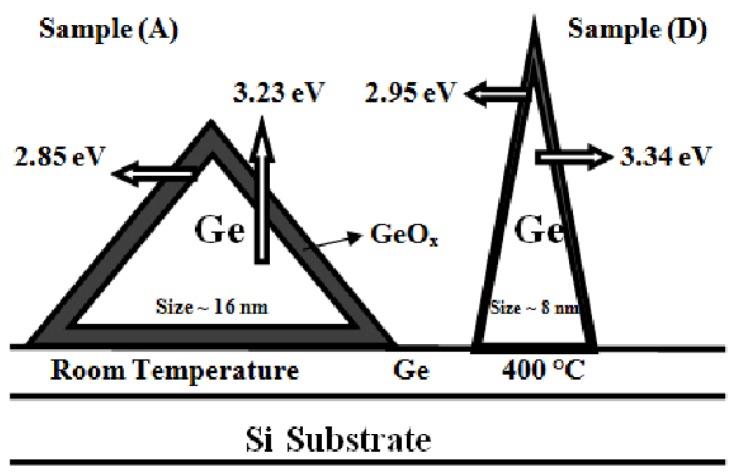
Schematic diagram of S-K growth mode of Ge QDs on Si substrate at two different substrate temperature for sample A (RT), D (400 °C) responsible for the origin of PL peaks presented in Figure 9.

Figure 1 shows the energy dispersive X-ray (EDX) spectra of the sample D to confirm the presence of Ge in addition to the silicon, the oxygen, and the carbon. The occurrences of Ge peaks confirm the existence of nanoislands composed purely of Ge as observed in the AFM images (Figure 5). The appearance of the oxygen peak is due to the passivation of the surface dangling bond under exposure of atmospheric oxygen. The FESEM images of the samples A and D (Figure 2a,b) clearly shows the presence of high density Ge QDs. The particle sizes (~8 nm to ~17 nm) estimated from FESEM are in conformity with the AFM. A schematic illustration of the pyramidal QDs structure based on structural and optical analyses is presented in Figure 3. The surface of the Ge QDs is modeled as covered by thin oxide layers having a thickness of a few nm, and the Ge islands grown at RT are considered to have the thickest oxide layer.

### 2.2. XRD Spectra

**Figure 4:**
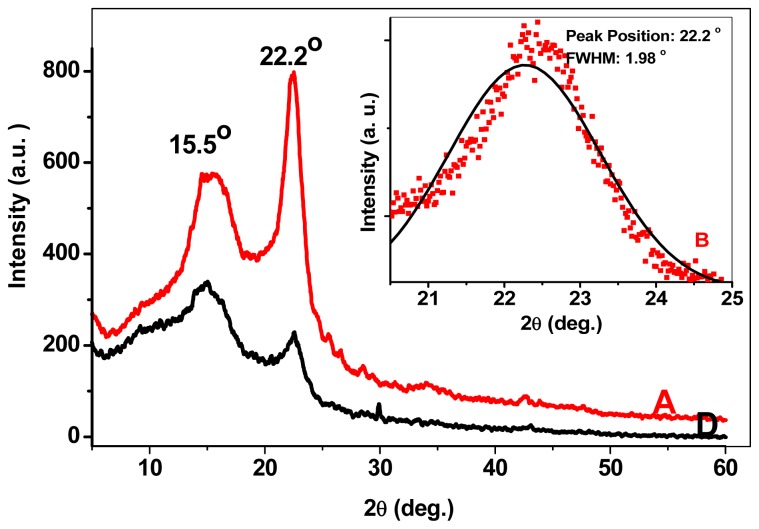
XRD spectra of samples A and D. The inset shows the de-convolution (Gaussian function) of the strongest peak at 2*θ* ~ 22.2°.

The XRD spectra for the 200 °C substrate temperature (lower curve) and 400 °C substrate temperature (upper curve) samples are shown in figure 4. Two prominent peaks associated with GeO_2_ core-shell structure and Ge QDs are evidenced. The strongest peak is related to Ge QDs located at 22.2° [14] whose full width at half maximum (FWHM) is 1.98° (inset). The broadening of the peak describes the quantum size effect of the nanoislands. Upon decreasing the substrate temperature, the peaks become sharper and the FWHM of each peak decreases indicating the increase of average QDs size. The average size of Ge QDs estimated using Scherrer formula is ~8 nm.

### 2.3. AFM Analysis

**Figure 5:**
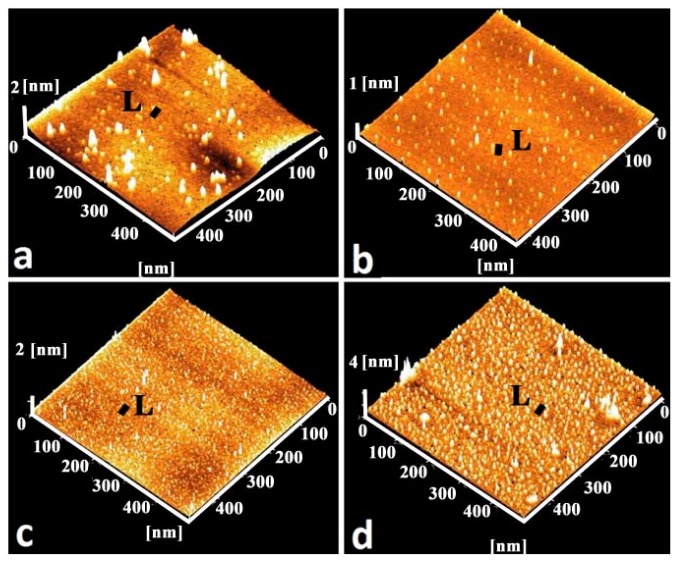
3D atomic force microscopy (AFM) images of sample A (**a**), B (**b**), C (**c**) and D (**d**).

A typical AFM image of Ge/Si(100) QDs at different growth temperatures is shown in Figure 5. The line scan (*L*) profile of the samples (Figure 7e–h) is also illustrated. The samples consist of mono-modal distribution of pyramidal shaped islands. The surface clearly exhibit well-resolved regular topography of germanium particle with ultra small size as evidenced from three-dimensional AFM. The estimated average size (number density) for the samples A, B, C and D are ~15 nm (2 × 10^3^ μm^−2^), ~8 nm (8 × 10^3^ μm^−2^), ~8.5 nm (14 × 10^3^ μm^−2^) and ~8 nm (14 × 10^3^ μm^−2^) respectively. The effect of size reduction of Ge islands with increasing substrate temperature from room temperature to 400 °C can be explained in terms of different kinetic mechanism. While uniformity is increased at higher growth temperatures, self-ordering observed in the super-lattice structures may be required to achieve the QDs density and the degree of uniformity needed for their architectures. Undoubtedly, the increase of the growth temperature leads to a high degree of intermixing via thermal diffusion. This in turn lowers the strain energy that favors nucleation on subsequent islands. At a higher substrate temperature sample (A), the Ge adatoms will diffuse to a longer distance at surface and prefer to produce new nucleation centers with narrow size distribution. A small pyramidal structure that requires higher energy for activation due to entropy maximization would appear.

#### 2.3.1. Size Distribution

**Figure 6:**
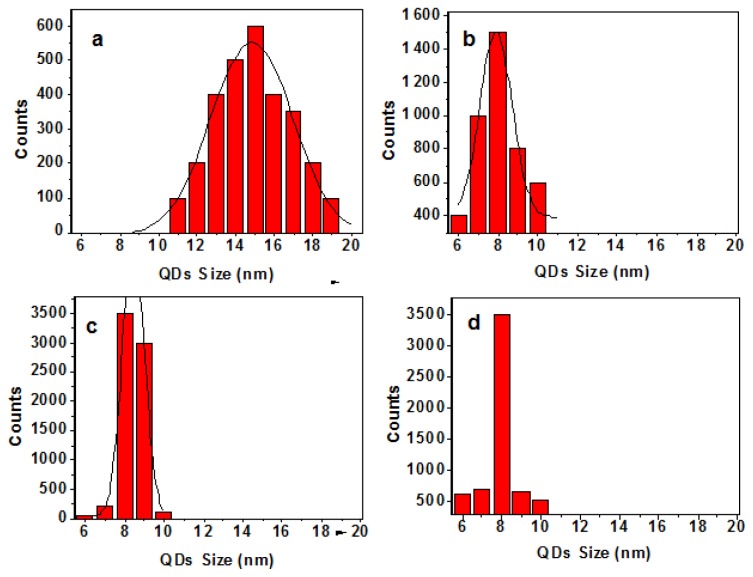
Size distribution of samples A (**a**), B (**b**), C (**c**) and D (**d**).

**Figure 7:**
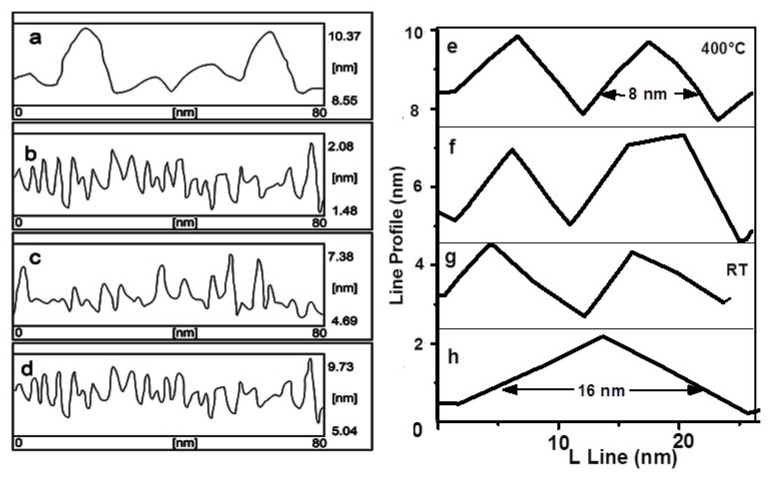
Height fluctuation of samples A (**a**), B (**b**), C (**c**) and D (**d**), line scan profile of sample A (**e**), B (**f**), C (**g**) and D (**h**).

Results for the substrate temperature dependent size distribution are illustrated in Figure 6. The island size is found to decrease from ~16 nm to ~8 nm with a decrease of FWHM thereby produced the narrow size distribution as the substrate temperature increase from RT to 400 °C. Results for the roughness fluctuation in relation to the variation in the height distribution of the islands corresponding to the samples A, B, C and D are presented in Figure 7a–d respectively. The roughness variation is quite robust and smooth at 400 °C and is expected because the island distribution has a regular pattern. However, as the substrate temperature is decreased the larger particles are formed and thereby resulted in irregular fluctuation of the height distribution, as indicated clearly in the AFM micrograph.

#### 2.3.2. RMS Roughness

**Figure 8:**
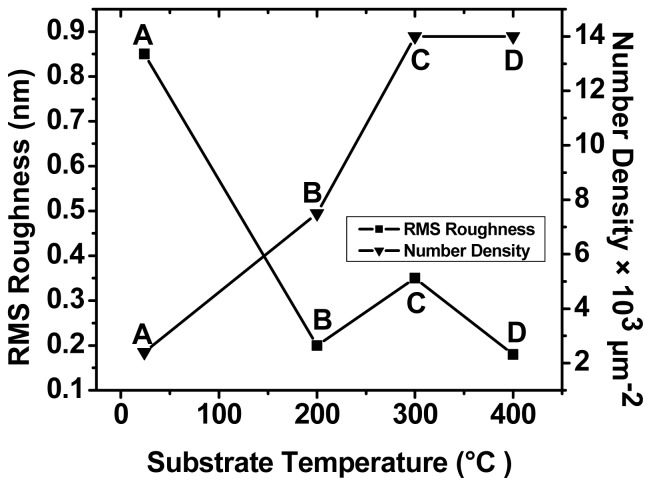
Root mean square (RMS) roughness and number density of samples.

For determining the sample quality, which eventually decides the optical behavior, the scattering of light and the nature of the surface are important factors. The RMS roughness is a measure of these properties [8]. The variation of RMS roughness and number density as a function of substrate temperature is shown in Figure 8. The corresponding fluctuation in the height distribution (Figure 8) expressed in terms of RMS roughness shows a monotonic decrease with substrate temperature.

It is clearly seen that beyond 300 °C, little change either in the number density (Figure 8) or in the roughness is evident. This observation has a direct correlation with the height of the peak in the corresponding normalized distribution obtained from the AFM images. The results obtained might provide a method to control the morphology of QDs, which could be used for tuning the narrow size distribution and ultra small size Ge nanoparticle.

### 2.4. Photoluminescence Results

**Figure 9:**
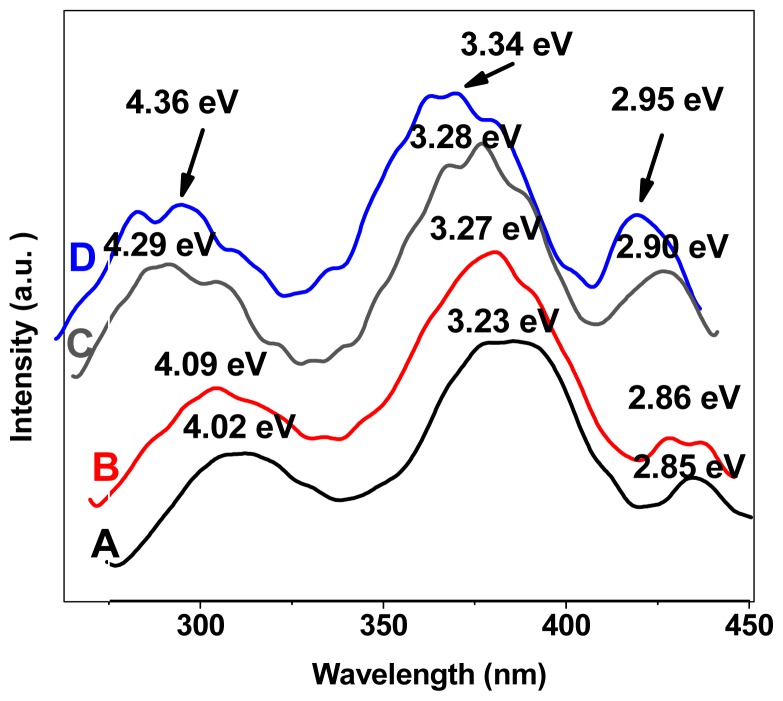
Photoluminescence spectra of samples A, B, C and D.

The room temperature PL spectra for four samples A, B, C and D are depicted in Figure 9. Three peaks appearing at approximately ~2.85 eV, 3.23 eV and 4.02 eV, indicating the interaction between Ge, GeO*_x_*, the possibility of the formation of a core-shell structure for the Ge QDs, and probably other kind of nanostructures with different symmetries. The role of surface passivation by atmospheric oxygen is important as it strongly affects the optical behavior. After the deposition, the Ge QDs may be encapsulated by the oxygen layer to form a GeO*_x_* layer, thereby resulting in the formation of the core-shell structure. Configuration of core-shell structures in different thickness and sizes gives rise to a peak and shift in the PL. At the nanoscale, the blue shift in the HOMO-LUMO transition energy gap becomes prominent. The quantum size effect drives the visible PL shift by changing the band gap nature from indirect to direct [15]. The increase in substrate temperature causes the formation of smaller islands; the mix state of Ge and the thinner GeO*_x_* interface reaction give rise to a PL peak at around ~2.86 eV, 2.90 and 2.95 eV for samples B, C and D, respectively.

Four intense peaks around ~3.23 eV, 3.27 eV, 3.28 eV and 3.34 eV are clearly seen for samples A, B, C and D, respectively, which are attributed to the presence of Ge QDs. The red shift (~0.11 eV) of the PL peak position for different particle size, which is due to the different substrate temperature, is in close agreement with observations made by others [11,16]. The island-size-dependent shift in the PL peak position at different annealing temperatures is attributed to quantum confinement effects. The maximum PL intensity is obtained for sample D with larger number density, which is explained in terms of the generation of a larger number of photo-carriers that contribute to the emission cross section. Our results confirm that the formation of core-shell structures, the presence of mix states, the quantum size, and surface effects are responsible for the visible luminescence in Ge nanostructures. The weak peak at ~2.95 eV observed in sample D presumably originates from the Ge and GeO*_x_* interface. The inner core of these Ge islands consists of finely distributed nanoparticle, and the peak at 3.23 eV may originate from these nanostructures in sample A. The optical properties, the narrow size distribution of nontoxic germanium nanocrystals and quantum confinement effect reported here can make them excellent candidates for future biological (especially biomedical) applications.

## 3. Experimental Section

Ge QDs are fabricated in the vacuum chamber pumped with diffusion and rotary pumps at a pressure of ~10^–3^ Pa. The Ge disc (purity 99.99% and 3 inches in diameter) is used as a target. The measured temperature on the rear face of the substrate holder during deposition is room temperature (RT) (sample A), 200 °C (sample B), 300 °C (sample C), and 400 °C (sample D). The radio frequency power, Ar flow, and deposition time for all samples are 100 W, 10 Sccm and 180 s respectively. Before loading the substrates in the sputtering chamber, the oxide layers from the substrate surface are removed by dipping the samples in weak hydro fluoric acid (HF ~5%), then by ultrasonic bath at room temperature for 20 min and finally dried by blowing nitrogen over them. Atomic force micrographs are recorded and the room temperature PL measurement is performed in the visible region using an excitation wavelength of 239 nm. The elemental composition of the Ge QDs is measured by energy dispersive X-ray (EDX) diffraction. The x-ray diffraction (Bruker D8 Advance Diffractometer) using Cu-Kα radiations (1.54 Å) at 40 kV and 100 mA are employed. The 2*θ*range is 0–60 with a step size of 0.021 and a resolution of 0.011.

## 4. Conclusions

Ge QDs with precise size and shape distribution are synthesized using rf magnetron sputtering technique. The influence of substrate temperature on the surface morphology and photoluminescence response of such QDs is studied. The occurrence of narrow size distribution and light emitting behavior in the visible region confirm their potential application in nanophotonic and optoelectronic applications. The observed RMS roughness and the number densities are found to be highly sensitive to the substrate temperature. We were able to ascertain the optimum growth conditions for TS = 400 °C, Ar flow = 10 Sccm, rf power = 100 W and deposition time = 180 s. The blue shift ~0.11 eV of the intense PL peak with decreasing nanoparticle size is attributed to the quantum confinement and surface passivation effect. However, transferring these non-toxic QDs into aqueous media and capping them is worth further research. Our method of fabrication is easy and economic.
